# Increase in Tuberculosis Cases among Prisoners, Brazil, 2009–2014^1^

**DOI:** 10.3201/eid2303.161006

**Published:** 2017-03

**Authors:** Paul M. Bourdillon, Crhistinne C.M. Gonçalves, Daniele Maria Pelissari, Denise Arakaki-Sanchez, Albert I. Ko, Julio Croda, Jason R. Andrews

**Affiliations:** Yale University, New Haven, Connecticut, USA (P.M. Bourdillon, A.I. Ko);; Federal University of Grande Dourados, Dourados, Brazil (C.C.M. Gonçalves, J. Croda);; University of São Paulo, São Paulo, Brazil (D.M. Pelissari);; Ministry of Health of Brazil, Brasília, Brazil (D.M. Pelissari, D. Arakaki-Sanchez);; Oswaldo Cruz Foundation, Campo Grande and Salvador, Brazil (J Croda, A.I. Ko);; Stanford University, Stanford, California, USA (J.R. Andrews)

**Keywords:** tuberculosis, TB, prisons, prisoners, Brazil, Mycobacterium, tuberculosis and other mycobacteria, bacteria

## Abstract

During 2009–2014, incarceration rates in Brazil rose 34%, and tuberculosis (TB) cases among prisoners rose 28.8%. The proportion of national TB cases that occurred among prisoners increased from 6.2% to 8.4% overall and from 19.3% to 25.6% among men 20–29 years of age.

In 2014, Brazil had the world’s fourth largest prison population, with more than half a million prisoners in 1,424 facilities (*1*). Prison conditions include overcrowding, frequent prisoner movement, poor ventilation, and limited access to diagnostic facilities; these conditions favor tuberculosis (TB) transmission (*2**–**5*). Many key factors for progression from TB infection (determined by positive test results) to disease (determined by clinical diagnostic criteria) also are common; these include alcohol and drug abuse, tobacco smoking, undernutrition, and HIV prevalence (*6*). Although surveys have reported high rates of TB among prisoners in Brazil (*6**–**9*), state-level and temporal trends in TB notifications and the contribution of the epidemic in prisons to the overall TB case burden have not been evaluated. To estimate trends in TB cases among prisoners and to identify incarcerated populations at high risk for infection, we analyzed data from the national disease notification database, census data, and administrative data.

## The Study

We obtained individual-level data on TB cases reported during 2007–2014 in the Brazil national notification database (Sistema de Informação de Agravos de Notificação; SINAN). A new form for SINAN was introduced in 2007, which led to an increase in documentation of incarceration status from 68% in 2007 to 94% in 2009. Our analysis therefore focuses on data from 2009–2014, when documentation rates were consistently >92%. We extracted demographic data on incarceration status, sex, age, and state as well as clinical presentation, HIV status, and sputum smear positivity. This work was approved by the human research protection program at Yale University and the institutional review board at the Federal University of Grande Dourados.

Among prisoners, TB case-patients were defined as persons for whom TB was notified while they were incarcerated, including those in penal institutions awaiting trial or sentencing. To calculate annual TB notification rates (cases per 100,000 persons), we used population estimates for Brazil from the 2010 Census and projections and mid-year incarcerated population data from the Ministry of Justice. To compare TB notification rates between incarcerated and nonincarcerated populations, aggregated at the state level, we used the Pearson product-moment correlation.

During 2009–2014, a total of 38,327 (7.3%) of the 526,569 cases of TB reported in Brazil occurred in prisoners. The total number of cases reported in prisoners increased 28.8%, from 5,556 to 7,157 per year, and the proportion of cases notified among prisoners increased from 6.2% in 2009 to 8.4% in 2014. During the same period, the prison population grew from 409,287 to 579,781, an increase of 41.7%, as overall incarceration rates rose 33.9%, from 214 to 287/100,000 persons. Because of that increase, the rate of TB notifications dropped from 1,357 to 1,234/100,000 for prisoners (9.1% decrease); meanwhile, the rate of TB notifications for nonprisoners dropped 12.2% (from 43.7 to 38.4/100,000 persons). Overall, the mean annual notification rate among prisoners was 31.3 times that of the general population (Table). 

**Table:** Annual average data for tuberculosis notifications, by incarceration status and gender, Brazil, 2009–2014

Category	General Population				Prisoners			p value, men vs. women*
All	Men	Women	All	Men	Women
Population, thousands	194,898	96,546	98,352		490	459	31	<0.0001
Annual no. cases	81,370	53,138	28,227		6,388	5,869	519	<0.0001
Notification rate, cases/100,000 population	42	55	29		1,307	1,281	1,703	0.001
HIV status reported, %†	66.1	66.6	65.3		65.9	65.9	66.0	0.97
HIV co-infection, %‡	17.4	18.2	15.8		15.9	15.2	24.1	0.003
Smear positive, %	55.5	57.2	52.2		65.5	66.4	55.2	<0.0001
Extrapulmonary, %	17.5	16.4	19.5		8.2	7.4	16.7	0.0002
Treatment success, %	66.6	64.4	70.9		69.5	69.8	66.0	0.09

*p values calculated with the Welch *t*-test comparing prisoners’ annual data by sex. †HIV status based on a positive or negative status result or AIDS diagnosis from the time of tuberculosis notification. ‡% HIV co-infection calculated as percentage positive HIV+ results among those with HIV status reported.

HIV status was known for 66% of all TB patients; prevalence was 15.9% among prisoners and 17.4% among nonprisoners (Table). Notification rates varied substantially among states. Average annual 2009–2014 notification rates among prisoners ranged from 225 (Rondônia) to 2,548 (Rio de Janeiro). At the state level, 2014 notification rates were positively correlated (Pearson ρ 0.54; p = 0.002) between the nonincarcerated population and prisoners.

**Figure 1:**
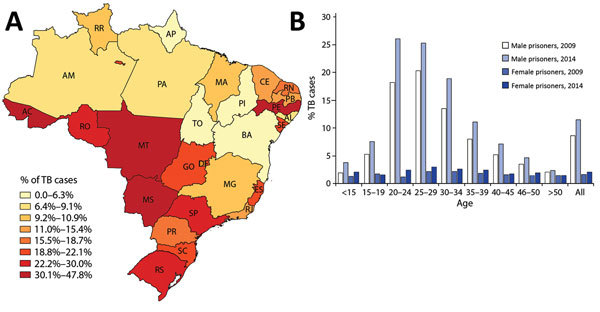
Proportion of tuberculosis (TB) cases among prisoners in Brazil, 2009–2014. A) Geographic distribution by state of the proportions of all TB cases diagnosed among male prisoners ages 20–29. Prisoners comprised 0–47.7% of all TB cases in this age group, with highest rates at the western border of Brazil. B) Sex and age distribution of the proportions of all notified TB cases diagnosed among prisoners in Brazil for 2009 compared with 2014. Prisoners of both sexes represent an increasingly disproportionate percentage of notified cases in all age groups, and male prisoners 20–29 of age represented >25% of cases among the age group in 2014. AC, Acre; AL, Alagoas; AP, Amapá; AM, Amazonas; BA, Bahia; CE, Ceará; DF, Distrito Federal; ES, Espírito Santo; GO, Goiás; MA, Maranhão; MT, Mato Grosso; MS, Mato Grosso do Sul; MG, Minas Gerais; PR, Paraná; PB, Paraíba; PA, Pará; PE, Pernambuco; PI, Piauí; RJ, Rio de Janeiro; RN, Rio Grande do Norte; RS, Rio Grande do Sul; RO, Rondônia; RR, Roraima; SC, Santa Catarina; SE, Sergipe; SP, São Paulo; TO, Tocantins.

**Figure 2:**
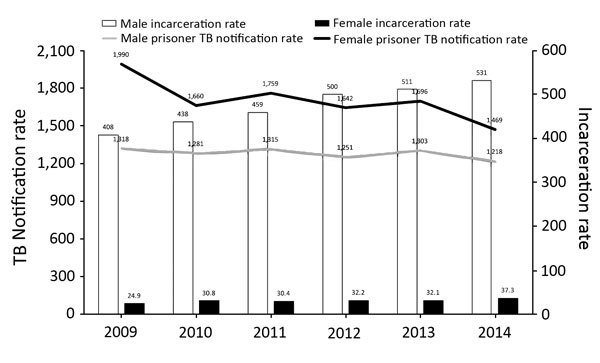
Temporal distribution of incarceration rates (prisoners/100,000 population) and TB notification rates (cases/100,000 prisoners), by sex, Brazil, 2009–2014. There was an appreciable increase in incarceration rates among men (30.2%) and women (50.0%) from 2009 to 2014. Although the incarceration rate of men averaged >15 times that of women, the average TB notification rate for women was higher (1,703 vs. 1,281/100,000 prisoners). TB, tuberculosis. AC, Acre; AL, Alagoas; AP, Amapá; AM, Amazonas; BA, Bahia; CE, Ceará; DF, Distrito Federal; ES, Espírito Santo; GO, Goiás; MA, Maranhão; MT, Mato Grosso; MS, Mato Grosso do Sul; MG, Minas Gerais; PR, Paraná; PB, Paraíba; PA, Pará; PE, Pernambuco; PI, Piauí; RJ, Rio de Janeiro; RN, Rio Grande do Norte; RS, Rio Grande do Sul; RO, Rondônia; RR, Roraima; SC, Santa Catarina; SE, Sergipe; SP, São Paulo; TO, Tocantins.

Men comprised 93.8% of the prison population and accounted for 91.9% of prison TB cases; 74.7% were 18–39 years of age, compared with only 39.8% in this age range among nonprisoners with TB. In 2014, a total of 25.6% of all men 20–29 years of age were prisoners (state range for all prisoners 0%–47.8%; Figure 1, panels A, B). Although there were far fewer female prisoners, incarceration rates rose faster for women (50.0% vs. 30.2%; Figure 2), and the average annual TB notification rate was higher for female prisoners than for male prisoners (1,703 vs. 1,281; Figure 2). The prevalence of extrapulmonary disease among women was twice that in men (16.7% vs. 7.4%; p = 0.0002). HIV co-infection rates were also higher for female prisoners (24.1% vs. 15.2%; p = 0.003, Table). 

## Conclusions

Prisoners have been recognized as a group at high risk for TB, but there has been limited assessment of the contribution of recent increases in TB prevalence in the prison population to the overall TB case burden in Brazil. We combined data from a national TB notification database with incarceration data and found that prisons account for a growing proportion of the national TB burden in Brazil. In 2014, a total of 8.4% of all reported cases occurred among prisoners, who represent <0.3% of the population, representing a 35.4% increase in the proportion of TB cases occurring among prisoners in 5 years. Among young men 20–29 years of age, a growing proportion (>25%) of TB cases occurred among those who were incarcerated. For the nonincarcerated population, TB notification rates fell by 12.2% during this period; however, when prisoners were included in assessments, 16.8% of these gains were offset by increasing incarceration rates, as notification rates among prisoners were 31.3 times higher than those for the general population. Failure to address TB in an expanding incarcerated population may be a critical barrier to achieving national targets for TB control.

Our analysis also revealed that female prisoners are at higher risk for TB. Incarceration rates among women rose faster, and TB notification rates and HIV co-infection rates were higher than corresponding rates among men. This finding is in contrast to the overall global epidemiology of TB and to national data for the nonincarcerated population, which demonstrated higher TB notification rates among men than women (50.1 vs. 26.5/100,000 persons; Table). A potential explanation is the higher rate of HIV-associated TB among female prisoners than among men (24.1% vs. 15.2%). We previously reported a higher prevalence of HIV among female prisoners in Brazil (*10*), as have others (*11**,**12*). HIV alone is unlikely to explain the large disparities in TB rates between male and female prisoners; more data are needed to explain the inverted sex disparities (compared with the general population) in TB patients in prisons in Brazil.

This study had several limitations. First, we assessed notification rates at the national and state level; although we observed substantial heterogeneity at the state level, more data on prison characteristics and incarceration rates are needed to understand factors driving TB in prisons. In particular, the relative contribution of individual risk factors for TB reactivation and the effects of prison environments and policies on transmission are poorly understood. Second, our use of data from reported cases may include demographic or diagnostic misclassification. Third, we could not assess cases among recently released prisoners; many prisoners are released without sentencing or after serving sentences of <5 years (*1*), and infections acquired in prison may be diagnosed after prisoners are released. Consequently, the number of cases notified among prisoners (incarcerated at the time of diagnosis) probably underestimates the true effect of prisons on the population-level TB case burden.

Our findings demonstrate that the epidemic of TB in prisons represents a larger challenge to national TB control in Brazil than the threat posed to prisoners’ health (*4**,**13*). There is an urgent need to address notification rates in prisons in Brazil by using effective interventions such as active case detection, preventive therapy, and transitional care for released prisoners (*2**,**3**,**5**,**14*). A recent study demonstrated high rates of nonadherence to treatment among prison prisoners in Brazil, underscoring the need to ensure that prisoners complete treatment to prevent transmission within and outside prisons (*15*). In addition to improving access to TB diagnosis and treatment within prisons, addressing rising incarceration rates and improving living condtions that favor transmission in prisons may be critical to combat TB among prisoners (*16*).
